# Promises and potential pitfalls of long-acting injectable pre-exposure prophylaxis

**DOI:** 10.4102/sajhivmed.v24i1.1497

**Published:** 2023-07-27

**Authors:** Carey Pike, Elzette Rousseau, Linda-Gail Bekker

**Affiliations:** 1 Desmond Tutu HIV Centre, Faculty of Health Sciences, University of Cape Town, Cape Town, South Africa

**Keywords:** HIV prevention, pre-exposure prophylaxis, long-acting PrEP, Cabotegravir long-acting, injectable PrEP

## Abstract

The number of products that can provide pre-exposure prophylaxis (PrEP) for HIV prevention is expanding, with three now approved in South Africa (oral Tenofovir-based PrEP, injectable Cabotegravir, and a Dapivirine-based vaginal ring) and more in the development pipeline. Although highly effective and safe, oral PrEP products have not reduced HIV incidence in South Africa to the extent seen in other countries, primarily due to adherence challenges, rapidly diminishing persistence over time, and insufficient scale-up of PrEP service delivery. The Dapivirine vaginal ring, which provides 1-month-long protection, provides women with a new and discreet choice for PrEP; however, it is Cabotegravir long-acting (CAB LA) that is anticipated to land the largest impact. Administered as an intramuscular injection given every 2 months, CAB LA is safe, highly efficacious, and expected to become available in South Africa in late 2023. Yet, clinical and implementation questions remain, including the need to understand and characterise breakthrough HIV infections amongst CAB LA users, knowledge of how to package each PrEP product in a new context of PrEP choice, and how to avoid the remedicalisation of PrEP access following extensive efforts to make oral PrEP delivery differentiated and community based.

**What this study adds:** A new HIV prevention product, injectable Cabotegravir long-acting (CAB LA) for pre-exposure prophylaxis (PrEP), has been approved for use in South Africa. This article explores CAB LA’s promise to boost HIV prevention product uptake and persistence and outlines the current implementation questions and concerns.

More than 40 years after the discovery of HIV and AIDS, one of the last remaining HIV vaccine candidates in late-stage clinical trials (MOSAIC trial) failed to show efficacy in preventing HIV infection compared to a placebo.^1^ In the continued absence of an effective HIV vaccine, alternative HIV prevention approaches remain critical to stemming the ongoing high annual incidence of new HIV infections.^2^ Although the annual HIV incidence has decreased by 54% since its peak in 1996, in 2021 an estimated 1.5 million people were infected. In sub-Saharan Africa (SSA), women and girls account for the majority (63%) of all new infections, while elsewhere key populations face significantly higher risk (28 times greater risk amongst men who have sex with men [MSM]; 35 times higher among people who inject drugs).^3^ Fortunately, there has been a flurry of new prevention products and interventions in the last 15 years, particularly the onset of antiretroviral-based prevention options.

## The promise and pitfalls of oral pre-exposure prophylaxis

When HIV emerged, HIV prevention strategies were restricted to the ABC strategy: abstinence, being faithful to your partner, and condoms. While an effective approach, this was viewed as unpalatable by most and unfeasible by many, and failed to stem the tide of HIV infections that was to come. Over time, a range of new methods have been developed that are people-centred, respectful of choice and differences in lifestyle, and allow for combined behavioural and biomedical approaches. A key biomedical approach available on the market since 2012 has been oral pre-exposure prophylaxis (PrEP). Oral PrEP regimens, which provide prophylactic antiretroviral cover with either tenofovir/emtricitabine (TDF/FTC) or tenofovir alafenamide/emtricitabine have a strong safety profile and were found to be highly effective in diverse populations, including adolescents, MSM and serodiscordant couples.^4,5,6,7^ Oral PrEP appeared poised to deliver the promised land: tumbling HIV infections, a user-controlled method, and a simple regimen – albeit tedious in its daily repetition.

The promised land has been slow to materialise – at least here in SSA. While as of February 2023, PrEP Watch reported 792 434 PrEP initiations in South Africa, which is more than three times the national target, high uptake of oral PrEP alone does not necessarily equate to effective HIV prevention and transmission reductions, as good adherence and long-term persistence are required to ensure optimal coverage of HIV exposure events.^8^ A global systematic review and meta-analysis that included 43 917 participants showed that 41.0% (95% confidence interval [CI]: 18.8–63.5) of participants discontinued PrEP within 6 months, with higher discontinuation rates (47%, 95% CI: 29.4–66.4) in the SSA region.^9^ The rate of return within the first month of PrEP initiation in South Africa, a checkpoint visit for side effects and adherence challenges, was found to be less than 30% (personal communication from the Western Cape Department of Health, August 2022). Although clinical efficacy trials showed high PrEP adherence among MSM, adherence challenges were forewarned during efficacy trials amongst African women (VOICE and FEM-PrEP), where efficacy was limited or completely absent.^10,11^ Drug level testing in these trials later showed that adherence to a daily PrEP regimen was achieved in less than 25% of participants, although effectiveness did improve in follow-up open-label trials.^10^ Pre-exposure prophylaxis use does need to be understood within the context of dynamic individual risk, however, where not all PrEP discontinuation is necessarily inappropriate. A recent study on PrEP discontinuation within a large, national programme in Kenya found that three-quarters of PrEP discontinuations occurred because participant risk profiles had changed (e.g. partner separation, partner viral suppression achieved) or they were using another appropriate HIV prevention method. Reaching people at times of high risk and allowing them to appropriately cycle on and off PrEP as needed may be critical, and realistic, for real-world success. In addition, for PrEP users who may not be exposed to HIV via the vaginal mucosa, such as MSM, the option of event-driven PrEP is now also feasible and effective.^12^ This is particularly useful for people in whom sex is reliably predictable, since it requires two pills 2 h – 24 h before sex and then a pill 24 h and 48 h later.^13^

**FIGURE 1:**
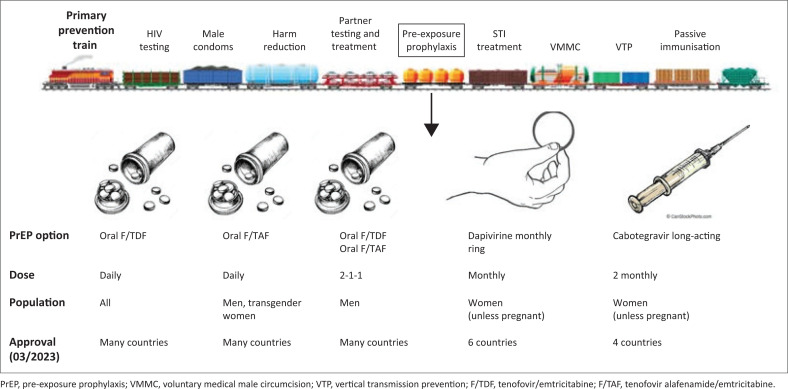
The primary prevention train: HIV prevention options, of which pre-exposure prophylaxis options are expanding to provide a choice of products that can be used preferentially by different populations.

Since the introduction of oral PrEP into the market, young people (< 30 years) have shown lower reported levels of adherence to oral PrEP agents.^14^ Reasons reported include clinical challenges such as side effects, logistical challenges such as pill storage and the risk of stock-outs, and social challenges such as fear and experiences of stigma, judgement, and violence.^14,15^ Preference and acceptability studies have indicated that a pill product may not be the preferred formulation in young South African populations. The ‘UChoose’ study employed a crossover design, where young women were randomised to receive contraceptive oral pills, rings, and injectable products analogous to potential new PrEP formulations, in order to determine user preference. The predominant reason provided by this cohort against daily oral contraception, and hence daily oral PrEP, was that three-quarters of participants feared they would forget to regularly take the tablets.^16^ The development of new longer-acting (less-frequently dosed) PrEP formulations, such as vaginal rings (the Dapivirine vaginal ring, which provides HIV protection for 28 days, was licensed for use in South Africa in March 2022), injectable products, and implants, have been proposed as a means to overcome these barriers and to maximise the potential of PrEP to prevent HIV among adolescents and young people (Figure 1).

## The promises and pitfalls of injectable pre-exposure prophylaxis

An injectable PrEP option, Cabotegravir long-acting (CAB LA) was approved by the South African Health Products Regulatory Authorities (SAHPRA) in December 2022 for use by individuals (weighing at least 35 kg) at risk of sexually acquired HIV-1 infection.^17^ Cabotegravir long-acting comprises Cabotegravir, a strand-transfer integrase inhibitor. When delivered as a suspension via a gluteal 600 mg intramuscular injection, it provides protection against HIV infection for at least 8 weeks.

This necessitates injections every 8 weeks, with an initial two injections 4 weeks apart.^17^ Two double-blind, double-placebo studies (HPTN 083 and HPTN 084) showed that CAB LA is superior to daily, oral TDF/FTC, as evidenced by a lower HIV incidence in the CAB LA arms compared to oral PrEP arms, in both male and female populations.^14,18^ This superior effectiveness likely resulted, at least partially, from improved adherence to injectable versus oral PrEP, as oral PrEP’s efficacy is up to 100% (95% CI: 83.7–100) when adherence is high (> 80%).^19^ HPTN 083 was conducted amongst cisgender men and transgender women who have sex with men in the United States, Latin America, Asia and Africa, and those in the CAB LA arm had a 66% lower risk of HIV infection compared to the TDF/FTC cohort.^18^ The HPTN 084 study was conducted amongst cisgender women across seven SSA countries and those in the CAB LA arm had an 88% lower risk of HIV infection compared to the TDF/FTC cohort.^14^ A systematic review and meta-analysis of all CAB LA safety and efficacy studies gave a pooled relative risk ratio of 0.21 (95% CI: 0.07–0.61), which indicates a 79% reduction in HIV risk with CAB LA compared to oral PrEP.^20^ Cabotegravir long-acting was subsequently approved for use as HIV PrEP in the United States, Australia, Zimbabwe and South Africa, and is recommended by the World Health Organization as an ‘additional prevention choice for people at substantial risk of HIV infection, as part of combination prevention approaches (conditional recommendation, moderate certainty of evidence)’.^21^

The predicted promise of CAB LA, according to a modelling study focused on CAB LA impact in SSA, is a reduction in HIV incidence by 29% over a 20-year period, with a knock-on reduction in AIDS deaths of 4540 annually, assuming an adult population of 10 million.^22^ This impact is at least partially attributed to an expected significant rise in PrEP use following CAB LA introduction, with 46% of those eligible for PrEP using compared to 28% in a no CAB LA scenario.^22^

This preference has also played out in the open-label choice component of the efficacy trials where the majority of participants are choosing the CAB LA option over oral PrEP. Part of the power of long-acting injectable PrEP modalities is thus to mitigate daily adherence challenges, while likely allowing for earlier detection of non-adherence due to the need for a healthcare professional to administer the drug directly.^15^ However, injectable PrEP also has the disadvantage of needing trained healthcare professionals to administer the product (a large volume, intramuscular injection), which requires PrEP users to return to health facilities every 2 months – a high clinic visit frequency compared to oral PrEP, which can be given as a 3-month supply.^23^ Alternative injectable formulations currently in clinical trials may offer a future solution. Lenacapavir for PrEP, currently in phase three trials known as ‘Purpose 1 and 2’, is administered subcutaneously every 6 months and so will also have the added advantage for possible self-administration, especially by at-risk populations familiar with injecting techniques (such as people who inject drugs and transgender populations already self-administrating gender-affirming hormone therapy).^23^ Lastly, while oral TDF/FTC PrEP is associated with a decline in bone mineral density, CAB LA has been found to be associated with a slight increase (0.82% with CAB LA use compared to −0.82% with TDF/FTC use, *P* < 0.01) over the short (57 weeks) and long term (105 weeks).^25^ This may make CAB LA a suitable alternative for people with low bone mineral density at PrEP initiation.

While CAB LA efficacy and safety have been broadly established, there remain a number of outstanding clinical and implementation questions.^21^ First, for women in SSA at high risk for HIV acquisition during pregnancy and breastfeeding (up to three times higher risk compared to non-pregnant women),^26^ PrEP provides a powerful means of protection, but safety for CAB LA injectable PrEP has not yet been established during pregnancy. No congenital birth anomalies were reported in HPTN 084, which reported a pregnancy incidence of 1.3 per 100 person years (0.9–1.7) and is currently in an open-label extension phase allowing for the inclusion of pregnant participants.^14^

Based on large numbers of pregnancies in HIV-treated women, oral TDF-based PrEP is now recommended in women at high risk of HIV acquisition before and during pregnancy and lactation. So far, data from animal and clinical trials have been reassuring, and injectable PrEP is not contra-indicated during pregnancy and lactation on the Patient Information Leaflet. However, given the paucity of safety data, healthcare providers are encouraged to counsel pregnant women on the risks and benefits of its use, and pregnancy registries are also being established.^17,27,28^ The Dapivirine vaginal ring has also recently been shown in early results from two studies from the Microbicides Trials Network to be safe for use in pregnant and lactating women, with one study showing very low concentrations of Dapivirine in infant plasma samples with good ring adherence, while the second study showed no safety concerns in the third trimester of pregnancy.^29,30^ The latter trial is ongoing to provide data regarding safety in early pregnancy (12–20 weeks). Overall, the results are showing promise that pregnant and lactating women will be able to safely access a range of PrEP options; however, close monitoring is ongoing and pregnancy registries remain good practice.

Second, Cabotegravir persists in the plasma for months following a single injection, only decreasing below the lower limit of quantification at a median time of 44 weeks for men and 67 weeks for women (primarily due to slower absorption in women, resulting in a longer half-life).^31,32^ Furthermore, there appear to be a number of factors that influence Cabotegravir half-life, including body mass index, indicating that it will likely be variable across populations.^32^ It remains unknown whether such residual drug concentrations (the pharmacological ‘tail’) following cessation of PrEP injections could contribute to selection of HIV variants resistant to integrase inhibitors. The development of integrase inhibitor resistance is concerning because it will render the individual unable to use Dolutegravir and other integrase inhibitors during treatment.^33^ Drug-resistant variants have been found in people who have been initiated onto CAB LA with an undiagnosed HIV infection and in the rare instances (< 0.3% in HPTN 083; 0.06% in HPTN 084) breakthrough infections have occurred while the individuals had high (protective) systemic concentrations of Cabotegravir.^34,35^ This may be due to false-negative HIV screening during acute HIV infection prior to seroconversion. In order to reduce this risk, the Food and Drug Administration has approved CAB LA injectable PrEP in the United States, with the proviso that initiation must include the use of a nucleic acid test. This requirement, if implemented in low- to middle-income countries, may be an additional cost and logistical barrier to injectable PrEP use. There was one recent case of a 28-year-old gender-diverse patient in the United States who seroconverted after 91 days on CAB LA, despite correct on-time dosing and laboratory monitoring for HIV infection.^36^ Such breakthrough infections have been rare in the case of oral PrEP (< 10 cases out of 2 million users, attributable primarily to viral resistance to TDF or FTC).^37^ These breakthrough infections have recently been coined ‘long-acting early viral inhibition (LEVI)’ syndrome in which the presentation of acute infection is atypical – people do not feel unwell, the viral load is reduced, and the infections are generally more difficult to detect with standard tests, resulting in very delayed diagnosis.^38^ The majority of these rare infections have shown resistance to integrase inhibitors and further studies, such as the PICASSO trial, are planned to guide what the optimal first-line treatment could then be for CAB LA users that develop resistance. A modelling study contextualised in SSA predicts integrase inhibitor resistance to rise to 12.1% (4.1% – 30.9%) following CAB LA introduction, compared to 1.7% (0% – 6.4%) in its absence.^22^ While no new infections occurring after cessation of CAB LA have shown resistance, there remains concern that it is theoretically plausible due to the prolonged ‘tail’ and dwindling plasma concentrations. While further studies are needed to understand the level of risk, this also needs to be viewed in context with the development of resistance in treatment of people living with HIV due to inadequate adherence and the overall benefit of CAB LA in preventing this by preventing new infections.^21^

Third, although CAB LA has been approved for adolescent populations in South Africa and elsewhere, the original CAB LA trials did not include participants under the age of 18. There are two ongoing sub-studies of HPTN 083-01 and 084-01 that are evaluating the safety, tolerability, and acceptability of CAB LA among adolescent female and male patients (< 18 years).^39,40^ Early findings from HPTN 084-01 have shown CAB LA to be safe, tolerable, and acceptable to adolescent (< 18 years) female patients in South Africa, Uganda and Zimbabwe.^41^ In SSA, adolescents and youth are at high risk of HIV acquisition, with adolescent girls and young women (15–24 years) accounting for 25% of all new infections, despite representing just 10% of the population.^3^ HIV incidence starts to rise from age 15–19 years and peaks between 20 and 24 years for adolescent girls and young women and other key populations in the region.^42,43^ This makes their inclusion in injectable PrEP roll-outs a significant priority, especially considering the strong evidence that this population, in particular, has struggled with adherence and persistence with oral formulations and has voiced a preference for injectable options.^14,15,16^ The Lenacapavir, PURPOSE 1 trial currently underway in African women includes both pregnant and adolescent women.^24^

Finally, the cost of CAB LA, which is currently pegged substantially higher than that of oral PrEP, has already been called out by providers, patients, and advocates as far too high to be sustainable for public sector roll-out in the South African and lower- to middle-income country context. Given the predicted advantageous impact of CAB LA on PrEP use, HIV incidence, and AIDS deaths in SSA, CAB LA would be cost effective in a scenario where long-acting PrEP is delivered at the same or no more than double the cost of generic oral PrEP. This modelling study also recommended the use of antibody-based rapid HIV testing in order to be cost effective.^22^ The cost of CAB LA currently lies > 185 times higher than the cost-effectiveness threshold (estimated to be $60.00 – $199.00)^44^ for middle-income countries. ViiV Healthcare has provided generic version access to 90 countries in the Medicines Patent Pool, which includes South Africa alongside all other African nations, but notably excludes other middle-income countries with similar gross domestic products.^45^ Beyond the cost of CAB LA product, the need for and cost of close monitoring and follow-up with CAB LA users, at least initially, in order to monitor for breakthrough HIV infections and resistance development, should be considered.

## Fulfilling the promises through choice

**FIGURE 2:**
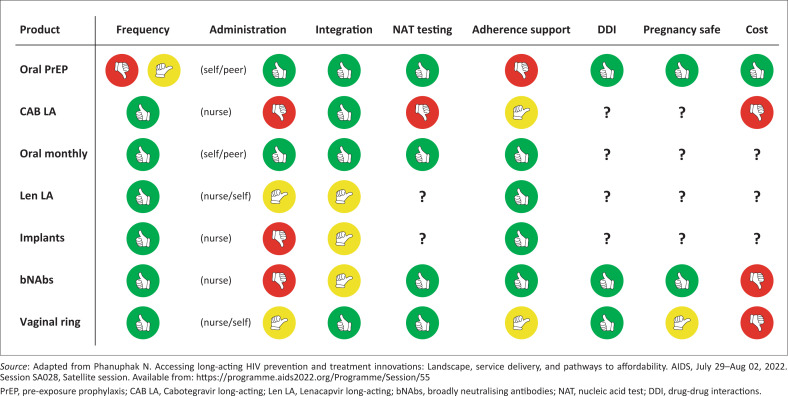
Will new pre-exposure prophylaxis (PrEP) options lead to remedicalisation? Comparison of implementation considerations across PrEP products (available and in clinical trials) and passive vaccination options.

Expanding PrEP choice needs to include more than a selection of PrEP options. There needs to be choice across all HIV prevention products, including condoms, voluntary male medical circumcision, education on treatment as prevention and U=U (undetectable viral load means there is no chance of sexual HIV transmission – it is untransmissible). Individuals’ preferences may cycle between options or include multiple approaches. There should be choice around where and how to receive PrEP, including health facility versus community-based delivery services. While there have been extensive efforts to diversify service delivery for oral PrEP, there are a number of anticipated challenges for delivering injectable products outside of a clinical environment (Figure 2).^46^ This may turn out to be a major affecter of choice, depending on whether someone would be happier accessing oral PrEP via courier or from a community depot versus accessing injectable from a formal clinic facility. Innovation to overcome the remedicalisation of PrEP with injectables is possible, especially off the back of coronavirus disease 2019 vaccine campaigns that included highly accessible pop-up vaccine centres and ‘Vaxi Taxis’.^47^ Alternatively, PrEP choice could further be couched into a menu of other sexual reproductive health services, such as contraception and testing, for sexually transmitted infections. Ultimately, choice should look like a fast-food menu: Would you like a side of condoms with your oral PrEP bottle? Upsize from oral to injectable PrEP! Spend 20 minutes extra and also receive contraceptive choice counselling from the same friendly healthcare provider.

There are a number of injectable PrEP and PrEP choice trials poised to commence in South Africa in 2023, some of which are poised to investigate uptake of CAB LA, the impact of CAB LA on PrEP persistence, patient and provider perspectives on PrEP products and delivery settings, and what a feasible and acceptable delivery package would look like for various populations.^48^ These efforts are necessary if we are to realise the full potential of CAB LA and PrEP choice, as well as set the ground for other longer-acting modalities.
